# The C677T methylenetetrahydrofolate reductase variant and third trimester obstetrical complications in women with unexplained elevations of maternal serum alpha-fetoprotein

**DOI:** 10.1186/1477-7827-2-65

**Published:** 2004-09-07

**Authors:** Natalie K Björklund, Jane A Evans, Cheryl R Greenberg, Lorne E Seargeant, Carol E Schneider, Bernard N Chodirker

**Affiliations:** 1 Department of Biochemistry & Medical Genetics, University of Manitoba, Winnipeg, Canada; 2 Department of Pediatrics & Child Health, University of Manitoba, Winnipeg, Canada; 3 Department of Community Health Sciences, University of Manitoba, Winnipeg, Canada; 4 Department of Obstetrics and Gynecology & Reproductive Sciences, University of Manitoba, Winnipeg, Canada

## Abstract

**Introduction:**

The C677T MTHFR variant has been associated with the same third trimester pregnancy complications as seen in women who have elevations of maternal serum α-fetoprotein (MSAFP). We hypothesized that these women with third trimester pregnancy complications and MSAFP elevations would have an increased frequency of the variant compared to an abnormal study control group (women with MSAFP elevations without pregnancy complications) as well as to normal population controls.

**Methods:**

Women who had unexplained elevations of MSAFP in pregnancy were ascertained retrospectively. The frequency of the C677T MTHFR variant among those women with unexplained elevations of MSAFP who had experienced later pregnancy complications was compared to that of women with unexplained elevations of MSAFP without complications as well as to that of the previously established Manitoba frequency.

**Results:**

Women who had complications of pregnancy and an unexplained MSAFP elevation had a higher allele frequency for the C677T MTHFR variant (q = 0.36,) compared to women with MSAFP elevations and normal pregnancy outcomes (q = 0.25, OR 1.73 95% CI 1.25–2.37, *p *= 0.03). The frequency was also higher than that of the population controls (q= 0.25, OR 1.70 95% CI 1.11–2.60, *p *= 0.007). The frequency in women with MSAFP elevations without pregnancy complications was not significantly different from that of the population controls (*p *= 0.41).

**Conclusion:**

Women with unexplained elevations of MSAFP and who experience complications in later pregnancy are more likely to have one or two alleles of the C677T MTHFR variant.

## Background

Significant elevations of amniotic fluid and maternal serum alpha-fetoprotein (MSAFP) have been shown to be associated with spina bifida and other neural tube defects (NTD). The province of Manitoba, Canada offers province-wide midtrimester MSAFP screening to all pregnant women. It has been recognized that some pregnant women with midtrimester unexplained elevations of MSAFP [1,2].

Increased total plasma homocysteine alters placental function and has been associated with the same complications that are associated with unexplained elevated MSAFP [3-7]. The C677T MTHFR variant has also been associated with complications of pregnancy in some, but not all, studies [8-10] C677T MTHFR may therefore contribute to complications of pregnancy by elevating serum homocysteine. Poor placental function could result in both an unexplained elevation of MSAFP and complications of pregnancy. We therefore hypothesized that women with third trimester pregnancy complications and MSAFP elevations (cases) would have an increased frequency of the variant compared to the Manitoba population (population controls) or women with MSAFP elevations without pregnancy complications (study controls) if they had low folate intake.

## Methods

### Background to methods

In a small pilot study of 32 couples, we found that women who had an unexplained elevation of MSAFP and a normal midtrimester fetal ultrasound, and their partners, had a significantly increased C677T MTHFR frequency compared to Manitoba newborns (RR 1.42, 95% CI 1.08–1.85, p = 0.012, two tailed) [11]. The newborn study that examined 977 anonymous consecutive neonatal screening blood spots showed that 36% of Manitoba newborns were heterozygous and 7% were homozygous for C677T MTHFR [12] (q = 0.25). Subsequently, on evaluation of the pregnancy outcomes of our pilot study women, we noted that, among eight women who had gone on to experience complications of pregnancy, the odds ratio for having the C677T MTHFR allele was 2.3 times higher than in the Manitoba population. However, the result was not statistically significant (*p *= 0.151, two tailed) indicating the frequency was increased but, this could have been a random result.

### Ascertainment and recruitment of study population

All pregnant women in Manitoba are eligible for routine serum screening through the voluntary MMSSP. In Manitoba, an elevation of MSAFP is defined as 2.3 multiples of the median (MOM) or greater. Candidates for inclusion in this study were women with an unexplained MSAFP elevation (i.e. not due to fetal anomalies, incorrect estimation of gestational age, previously unrecognized fetal demise, or multiple gestation) with either a complicated or uncomplicated pregnancy outcome. After appropriate approvals had been obtained from The University of Manitoba Health Research Ethics Board, review of the screening records began in 1999 and took three years. For a study using a two step consent to participate methodology administered by mail, the expected response rate (after excluding lost to follow-up) would be 20% [13]. Our goal was 1000 invitations. We anticipated this would result in approximately 120 participants. This would be double the minimum number of participants suggested by the power analysis we had conducted for the pilot study. To increase our response rate further, we added telephone follow-up for invited potential participants who were non-responders [14].

All screening records from 1995–1999 were reviewed, accounting for 783 invitations. Records for 2000–2002 were reviewed systematically as outcome information on each pregnancy became available to MMSSP. Records for 1990–1994 were then reviewed systematically in order to bring the total up to 1000. If a woman had more than one pregnancy with an elevation of MSAFP screened by the MMSSP, only the first pregnancy encountered in the retrospective review was used for the study. Previous or subsequent pregnancies were not included. Women with preexisting conditions known to influence pregnancy outcome, such as essential hypertension, and mothers of babies with major congenital anomalies were excluded. Eight women who had relinquished their babies for adoption or whose babies were placed in foster care were also excluded.

Women who met the inclusion criteria were divided into two groups for analysis. Cases were defined as women with pregnancies complicated by one of the complications previously shown to be associated with an unexplained elevation of MSAFP at midtrimester [2]. These include: intrauterine growth restriction (IUGR) (<10^th ^percentile), pregnancy induced hypertension, preeclampsia, eclampsia, postpartum hemorrhage, retained placenta requiring manual delivery, abruptio placenta, premature delivery (<36 weeks gestation or requiring specialized neonatal care for prematurity) and unexplained fetal demise. Study controls were women with normal outcomes which were defined as those with delivery at term ≥ 36 weeks gestation), no complications of pregnancy, a normal placenta and a healthy baby. Definition of complications was based on ICDC-9 codes in the MMSSP outcome charts for each patient [15] which are then confirmed later by chart review for all those with a positive MMSSP result. All women ascertained as having unexplained MSAFP elevations and who fit the inclusion criteria above, were invited by letter to participate. The previously reported newborn study provided population control group data [12].

### Study questionnaires

Women who agreed to participate in the study were mailed the appropriate questionnaires and blood requisitions. The questionnaire included a semi-quantitative food frequency questionnaire (FFQ) based on standard methodology but, modified to suit Manitoba residents and previously validated for this population by biochemical analysis during the pilot study [11,16]. The survey included questions on vitamin supplement intake to determine preconceptional or prenatal supplementation as well as current use of vitamins. Dietary intake of folate and folic acid from supplements, and intake of the cofactors B_12 _and B_6_, were calculated from the FFQ for intake both during pregnancy and at the time of the study. A correction of an additional 0.1 mg for folic acid fortification that began in Canada in 1998 was included for pregnancies that began after fortification [17]. FFQ analyses were performed with the researcher blinded as to the status of the mother.

### Laboratory analysis

Total plasma homocysteine, red blood cell folate, and serum folate were determined using established methodology [18,19]. Samples were processed on site with clotting and separation by spinning. Sera was stored at 4°C during shipping to the central laboratory and until processing. DNA was extracted from whole blood and C677T MTHFR genotyping was performed using previously established methodology [11,20,21]. Genotyping and biochemical analyses were performed also blinded.

### Statistical analysis

Chi-squared analysis (one tailed unless otherwise noted) was used for allele frequency. Comparisons of potentially confounding factors between the case group and the study control group were undertaken. Parametric data were analyzed with the Student's *t *test for difference between means with Bonferroni correction for multiple comparisons. Data not normally distributed were analyzed using the nonparametric Mann-Whitney Rank Sum Test. Linear regression was used to test the validity of the dietary survey. A multivariate analysis included age, smoking, maternal weight at the time of MSAFP testing, presence of C677T MTHFR, gender and weight of infant, biochemical parameters, and FFQ results for folate, B_12 _and B_6_, both at the time of the survey and for during the pregnancy was undertaken. In order to avoid convergence due to the large number of variables, the analysis was completed in subsets of six variables. Variables with the higher association scores from these analyses were then combined for further testing in various combinations using stepwise multiple linear regression. Also linear regression analysis of each continuous variable with genotype results was performed. Corrections for multiple comparisons were included. Software used was NCSS Statistical Systems for Windows [22].

## Results

### Participation rates

Nine hundred and ninety four women were identified as eligible (342 cases and 652 controls). Of the 590 women successfully contacted, 130 (22%) agreed to participate (56 cases and 74 controls). Four hundred and four women were lost to follow-up. Cases were more likely to choose to participate than controls and this difference was significant (1.5, *p *= 0.030). There was no difference in the proportions of cases and controls that were lost to follow-up (*p *= 0.157). We had anticipated a 20% response rate and we achieved 24%.

### Genotype results

Genotypes were available for 54 cases and 73 controls for this analysis. Results are summarized in Table 1. The allele frequency for the C677T MTHFR variant in the Manitoba population has been previously established to be q = 0.25. Women who had complications of pregnancy and an unexplained MSAFP elevation had a higher allele frequency for the C677T MTHFR variant (q = 0.36) compared to women with MSAFP elevations and normal pregnancy outcomes (q = 0.25, OR 1.73 95% CI 1.25–2.37, *p *= 0.03). The frequency was also higher than in the population controls (q = 0.25, OR 1.70 95% CI 1.11–2.60, *p *= 0.007). The frequency in women without pregnancy complications and MSAFP elevations (study controls) was not significantly different than that seen in population controls (*p *= 0.41).

**Table 1:** Comparison of allele frequency of C677T MTHFR between cases, study controls, and population controls.

**Subjects**	**C/C (%)**	**C/T (%)**	**T/T (%)**	Comparing to study controls* OR (95%CI)	Comparing to population* OR (95%CI)
**Cases N = 54**	21 (39)	27 (50)	6 (11)	1.73 (1.25–2.37) (*p *= 0.033)	1.70 (1.11–2.60) (*p *= 0.007)
**Study Controls N = 73**	40 (55)	30 (41)	3 (4)	~	0.98 (0.46–1.55) (*p *= 0.410)
**Population N = 977**	557 (57)	352 (36)	68 (7)	0.98 (0.46–1.55) (*p *= 0.410)	~

* χ^2 ^comparison of allele frequency (total T and C) in each group, one tailed. Cases: women with unexplained elevations of MSAFP who had subsequent complications of pregnancy, (C = 69, T = 39) Controls: women who had unexplained elevations of MSAFP and no subsequent complications (C = 110, T = 36) Population controls were 977 newborns (C = 1466, T = 488) [12]. C/C = normal type, C/T = heterozygous for thermolabile variant, T/T = homozygous for thermolabile variant.

The case and study control groups included women at various stages of their child bearing years. Only one woman recruited as a control subject had a previous or subsequent pregnancy with an unexplained elevation of MSAFP and complications. She was a heterozygote for C677T MTHFR. No case subjects had a previous or subsequent pregnancy with an unexplained elevation of MSAFP and a normal outcome, but four case subjects had had a previous or subsequent pregnancy with complications after an elevated MSAFP. If the case versus control classification had been based on whether or not a woman had *ever *had a pregnancy with an unexplained elevation of MSAFP followed by complications, the association would still be present when compared to the population control (q = 0.3636, OR 1.72, 95%CI 1.27–2.61, *p *= 0.0055).

### Biochemical results

Heterozygotes and homozygotes for C677T MTHFR had lower average values (r = 0.978, *p *= 0.019) for serum folate than those who did not have the variant. None of the women were deficient in either serum folate (defined as <7.0 nmol/L) or red blood cell folate (defined as <430 nmol/L RBC). There was no significant difference in mean homocysteine levels (Table 2).

**Table 2:** Comparison of the parametric characteristics of women with unexplained elevations of MSAFP according to those with and without complications of pregnancy

**Characteristic**	**Mean Cases (SD)**	**Mean Controls (SD)**	***p *value**
MSAFP result	2.78 (± 0.62)	3.16 (± 3.76)	0.398
weeks gestation	17.1 (± 1.61)	16.9 (± 1.40)	0.432
μg/folate/day in pregnancy^2^	1216 (± 915)	1010 (± 892)	0.206
μg/folate/day at time of study^2^	557 (± 341)	523 (± 498)	0.588
erc folate (nmol/L RBC)	1234 (± 289)	1208 (± 317)	0.632
serum folate (nmol/L)	32.3 (± 5.80)	32.3 (± 5.71)	0.956
serum homocysteine (μmol/L)	7.8 (± 2.26)	8.4 (± 2.80)	0.246
μg B_12_/day in pregnancy^2^	12.4 (± 5.37)	13.4 (8.31)	0.488
μg B_12_/day at time of study^2^	8.9 (± 12.26)	8.6 (± 10.83)	0.899
mg B_6_/day in pregnancy^2^	8.4 (± 9.69)	7.2 (± 9.01)	0.461
mg B_6_/day at time of study^2^	6.0 (± 13.35)	5.5 (± 11.10)	0.792
mother's age at delivery	31 (± 4.19)	30 (± 5.19)	0.251
mother's weight in Kg	76 (± 17.26)	69 (± 16.09)	0.013^1^

^1 ^This value is not significant after Bonferroni correction for multiple comparisons. See discussion. ^2^Data was skewed due to a small number of women in both groups taking large dose vitamin supplements. When these women were removed from the analysis the result remained nonsignificant.

### Validity of surveys

Seven cases and one study control declined to fill out their dietary surveys. Mean values were inserted in the multivariate analysis for these eight women. The validity of the dietary survey was demonstrated again for this study by linear regression analysis. Consistent with known homocysteine metabolism [23], a negative correlation existed between serum homocysteine and both red blood cell folate (r = 0.945 *p *= 0.0052) and serum folate (r = 0.932, *p *= 0.0001). Higher intake of dietary folate (including synthetic folic acid from supplements) as reported by the FFQ for the time of study was associated with higher serum folate (r = 0.941, *p *= 0.0001) and higher red blood cell folate (r = 0.949, *p *= 0.0166).

We did several checks to determine that the women were answering their surveys accurately. Comparisons of specific data items available in the women's MMSSP charts at the time of pregnancy with the data reported in the surveys showed excellent agreement for every item examined indicating women answered questions accurately. Women who reported smoking (as a quantitative value from 0–3 based on 1/2 packs/day smoked) showed a negative correlation with serum folate (r = 0.923, *p *= 0.0062) consistent with accurately reporting their smoking habits [24]. Based on the results of these tests of the validity of our surveys, we are confident that the information provided by our participants was accurate.

### Analysis of FFQ survey and MMSSP data

The ethnicity of the infants born to the case mothers (based on the ethnicity reported for the infants grandparents) was 84% Caucasian, 5% Aboriginal. Mixed ethnicity was reported for 11% of the infants with one parent Caucasian and the other parent Aboriginal, or rarely Black or Asian. The ethnic distribution was the same for controls and is typical for the Manitoba population [25,26]. There were also no significant differences between cases and controls with respect to their place of residence within the province (such as rural versus urban address).

There were no significant differences in dietary and supplemental intake of folate, B_12_, or B_6_, or in the biochemical parameters of case and control mothers. There was no difference in the percentage of cases and controls who reported taking prenatal vitamin supplements during pregnancy (37/48 cases and 55/72) or taking vitamin and/or folate supplements preconceptionally (17/48 cases and 25/72 study controls).

We attempted to divide our cases into smaller groups by type of pregnancy complication. We also separated isolated IUGR and IUGR associated with hypertensive disorders of pregnancy. Most of the groups lacked power for statistical analysis due to small numbers. However, normotensive women whose fetus had IUGR (N = 12) had a higher frequency of the C677T MTHFR variant compared to the population controls (q = 0.33, OR 2.58 95% CI, 1.78–3.73, *p *= 0.013). Homozygosity for the C677T MTHFR variant is associated with IUGR in women who do not take vitamin supplements according to one large study of Canadian women [10,27]. Our findings are in agreement with this result as only 3/12 women took supplements. We found this effect in a group of combined heterozygous and homozygous women.

There was a trend towards higher mean weight for mothers who had complications at the time of MSAFP test in the individual comparisons, but this was not significant after correction for multiple comparisons (Table 2 and 3). The multivariate analysis did not reveal any unexpected associations, but it did show the importance of maternal weight as a variable (r = 0.933, *p *= 0.024). This was also not unexpected given that some of the complications we were examining are associated with obesity [28]. Even after controlling for women's weight in the multivariate analysis, the higher frequency of C677T MTHFR among cases remained significant (r = 0.734, *p *= 0.0462). There was no association between weight and MTHFR status (r = 0.431, *p *= 0.679).

**Table 3:** Comparison of the nonparametric characteristics of women with unexplained elevations of MSAFP according to those with and without complications of pregnancy.

**Nonparametric Characteristics**	**Cases**	**Controls**	***p *value**
Location			
Winnipeg	44	63	0.854
southern city	3	2	
southern town	3	5	
southern rural	8	8	
northern city	2	1	
northern town	1	4	
northern rural	5	7	
ethnicity			0.972
Caucasian	52	66	
Aborginal	2	2	
Mixed Caucasian/Aboriginal	7	6	
Mixed Caucasian/Black	1	3	
Asian	1	1	
Mixed Caucasian/Asian	1	1	
Unknown	3	1	
diabetes in pregnancy	2/67	1/80	0.207
maternal smoking present (0 = nonsmoker, 1–3 = half pks/day increments)	0 = 47, 1 = 11, 2 or more = 9	0 = 60, 1 = 8, 2 or more = 12	0.721
gender of baby	27 females, 40 males	42 females, 38 males	0.352
parity = number of women	0 = 37, 1 = 20, 2 = 7, 3 or more = 3	0 = 40, 1 = 29, 2 = 7, 3 or more = 4	0.254
previous miscarriages	11/67	15/80	0.203
previous case pregnancy	1	0	0.967

## Discussion

Unexplained elevations in MSAFP are known to be associated with an increased risk for complications of pregnancy [2]. Others have reported that presence of the C677T MTHFR variant in pregnant women with low folate intake is associated with increased risk for pregnancy complications [2,29-31]. The unique finding of this study is an increase in the frequency of the C677T MTHFR variant among women with normal folate intake, who went on to have complications of pregnancy after an unexplained elevation of MSAFP (Table 1).

The lack of folate deficiency in this population was unexpected, given previous research which showed that 23.6% of Newfoundland and Labrador women are folate deficient at their first prenatal visit [32]. As our study was retrospective, we did not have data on levels during pregnancy. It has recently been shown that the C677T MTHFR variant does not affect maternal serum homocysteine levels in pregnancy among women who take prenatal multivitamins [8]. Also a recent prospective study shows that there is no difference in homocysteine levels at midtrimester between women who later develop preeclampsia and those who do not [33].

As is the situation with NTDs, lack of folate deficiency by current definitions in a non-pregnant woman may not indicate that her folate intake is adequate for pregnancy. This would especially be true for women with the C677T MTHFR variant. Reexamination of the current definition of what constitutes a normal biochemical result for folate intake for women of child bearing age should be undertaken to clarify this.

We suggest that the negative effects of the C677T MTHFR variant are more likely to occur in early pregnancy before women began taking prenatal vitamins because the majority of our study participants took prenatal vitamins, but only 35% took preconceptional supplements. We suspect that reduced methylation interfering with cell proliferation in the placenta as originally suggested by Eskes (2000) [3].

In conclusion, using a retrospective case/control study, we have found that women with unexplained MSAFP elevations who have complications in later pregnancy are more likely to have the C677T MTHFR allele. Our resultsdo not suggest that C677T MTHFR predisposes a woman to having an elevation of MSAFP level (as we did not compare the C677T MTHFR frequency in women with and without elevated MSAFP), but having one or more copies of this variant predisposes such screen positive women to having complications in later gestation. It remains to be seen if other risk factors can be identified which can more accurately define this high risk group.

## Authors' contributions

All authors participated in original study design except CS. CG, BC and CS all acted as principal investigators for funding. NB assisted with all grant proposals. NB undertook the review of the individual MSAFP files and MMSSP database searches, designed the dietary and family history surveys, classified cases and controls, acted as study coordinator handling all aspects of participant contact, and provided data analysis. All authors also participated actively with NB for various aspects of the study. CG provided MTHFR genotyping. LS provided biochemical analysis. NB drafted the original manuscript with assistance from BC. CG and BC handled ethics approval assisted by NB. All authors read and approved the final manuscript.
